# Medial–lateral versus lateral-only pinning fixation in children with displaced supracondylar humeral fractures: a meta-analysis of randomized controlled trials

**DOI:** 10.1186/s13018-023-03528-8

**Published:** 2023-01-16

**Authors:** Binbin Xing, Bin Dong, Xiaoling Che

**Affiliations:** Department of Traumatic Orthopedics, Yuncheng Central Hospital, Shanxi Medical University, No. 3690, Hedong East Street, Yanhu District, Yuncheng, 044000 Shanxi Province China

**Keywords:** supracondylar humeral fractures, Pinning fixation, Ulnar nerve injury, Meta-analysis

## Abstract

**Background:**

Supracondylar humeral fractures (SCHFs) are frequent in children, and closed reduction with percutaneous pin fixation remains the standard surgical treatment for displaced SCHFs. Two pinning configurations, medial–lateral crossed entry pinning (MLP) and lateral-only entry pinning (LP), are widely used, but which one is superior to another one is still debatable. This meta-analysis aimed to compare the efficacy and safety of both pinning fixation methods.

**Methods:**

Randomized controlled trials (RCTs) were searched on PubMed, EMBASE, Web of Science, Cochrane library and Google Scholar. Relative risk (RR) and mean difference (MD) with corresponding 95% confidence interval (CI) were calculated for radiographical outcomes, functional outcomes and complications.

**Results:**

A total of 19 RCTs comprising 1297 Gartland type II and type III fractures were included. MLP had a decreased risk of loss of reduction (RR = 0.70, 95%CI 0.52–0.94, *P* = 0.018) but a higher risk of iatrogenic ulnar nerve injury (RR = 2.21, 95%CI 1.11–4.41, *P* = 0.024) than LP. However, no significant difference was observed for incidence of ulnar nerve injury if applying a mini-open technique in MLP group (RR = 1.73, 0.47–6.31, *P* = 0.407). There were no differences between both groups in loss of carrying angle (MD = − 0.12, 95%CI − 0.39 to 0.16), loss of Baumann angle (MD = 0.08, 95%CI − 0.15 to 0.30), excellent grading of Flynn criteria (RR = 1.06, 95%CI 0.99–1.14, *P* = 0.102) and pin tract infection (RR = 0.92, 95%CI 0.50–1.70).

**Conclusions:**

MLP is more effective in maintaining fixation, while LP is safer with respect to ulnar nerve injury. MLP with a mini-open technique reduces the risk of ulnar nerve lesion and is an effective and safe choice.

**Supplementary Information:**

The online version contains supplementary material available at 10.1186/s13018-023-03528-8.

## Background

Supracondylar humeral fractures (SCHFs) represent the most common type of elbow fractures in children, which account for nearly 10% of all fractures and 70% of elbow injuries in pediatric population [1, 2]. Children are much susceptible to SCHFs, due to the weak metaphyseal sclerotin of the distal humerus and the thin structure of metaphyseal bone between the coronoid fossa and olecranon fossa. SCHFs are classified according to Gartland’s criteria [3]. Gartland type I fractures are stable, whereas Gartland type II and III fractures present varying degrees of displacement and angulation with complications, such as nerve palsies and cubitus varus [4]. In 2006, Leitch et al. [5] proposed rare supracondylar fractures with multidirectional instability as Gartland type IV fractures.

Closed reduction with percutaneous pin fixation is currently the standard treatment of Gartland type II and type III SCHFs in children. There are two common choices of pinning configuration, medial–lateral crossed entry pinning (MLP) and lateral-only entry pinning (LP) using two or three pins, for SCHFs [6]. However, there is yet no consensus on the superiority of crossed pinning or lateral pinning technique [7]. Theoretically, MLP technique confers better biomechanical stability than LP technique [8] but has a potential risk of ulnar nerve injury resulted from the placement of a medial wire [9, 10]. Conversely, LP is a less biomechanically stable construct despite an advantage of avoiding ulnar nerve injury [11].

Several meta-analyses comparing the efficacy and safety of crossed pin fixation versus lateral pin fixation in children with SCHFs have been published [7, 9–15]. Yet, the results are mixed. Some meta-analyses found comparable construct stability and functional outcomes between both pinning configurations and recommended lateral pinning as a result of decreased risk ulnar nerve injury [9, 14, 15]. Some did not prioritize the fixation techniques because of a less fixation stability of lateral pinning but a higher risk of ulnar nerve risk of crossed pinning [7, 12, 13]. The inconsistency may be caused by the inclusion of non-randomized controlled trials (non-RCTs), i.e., retrospective case–control or prospective cohort studies, that have a low evidence level or a small number of eligible RCTs. Here, we conducted an updated meta-analysis with more recently published RCTs, to compare the risk of ulnar nerve injury, fixation stability, and functional and cosmetic outcomes of MLP versus LP in children with SCHFs.

## Methods

### Literature search strategy and inclusion/exclusion criteria

This meta-analysis was performed in accordance with the Preferred Reporting Items for Systematic Reviews and Meta-Analysis (PRISMA) statement (Additional file 1) [16]. We searched randomized controlled trials comparing the treatment effect of MLP and LP for children with SCHF in various literature databases, including PubMed, EMBASE, Web of Science, Cochrane library and Google Scholar, from inception to April 2022. The following keywords and their combinations were used for literature search: “supracondylar fracture,” “humeral or humerus,” “kirschner or pinning,” and “children or pediatric.” There was no language restriction. Since Google Scholar search yielded a large number of unrelated publications, only the first 200 articles that were ranked by relevance were reviewed for the eligibility to our meta-analysis. Moreover, we reviewed the reference lists of included articles for additional eligible studies.

An eligible study should fulfill the following criteria: (1) enrolled children with Gartland type II or type III SCHF; (2) used closed reduction with percutaneous pin fixation; (3) randomly assigned patients to MLP and LP groups; (4) reported radiographic outcomes, functional outcomes or complications. Specifically, medial–lateral crossed pinning using the mini-open technique to avoid injury of iatrogenic ulnar nerve was allowed [17]. Studies were excluded if they were retrospective or prospective non-RCTs, included only Gartland type I fractures, or did not provide sufficient data. Reviews, meta-analysis and meeting abstracts were discarded. For studies with overlapping samples, only the one that had a larger sample size or was published more recently was included. The non-standardized, novel fixation method, such as Dorgan’s cross-pinning [18, 19], was also excluded.

### Outcomes

Radiographic outcomes included carrying angle, loss of carrying angle, Baumann angle, loss of Baumann angle, loss of humerocapitellar (HC) angle, loss of metaphysio-diaphyseal (MD) angle and loss of reduction. Functional outcomes included Flynn criteria scores, loss of elbow extension, loss of elbow flexion and loss of range of motion. According to the criteria of Flynn et al. [20], the functional outcome was graded as excellent based on carrying angle and elbow motion. Iatrogenic ulnar nerve injury and pin tract infection were reported as complications.

### Quality assessment

We assessed the quality of all included RCTs by using Cochrane collaboration’s tool for assessing risk of bias. The risk of selection bias (random sequence generation, allocation concealment), performance bias (blinding of participants and personnel), detection bias (blinding of outcome assessment), attrition bias (incomplete outcome data), reporting bias (selective reporting) and other bias was graded as low, high or unclear.

### Data extraction

The following information was extracted: first author, publication year, Gartland types of fractures, sample size, mean age, sex distribution, duration of follow-up, the number of events for categorical variables, and the mean value and standard deviation (SD) for continuous variables. The literature search and selection, quality assessment, and data extraction were conducted by two independent authors. Any discrepancy was resolved by full discussion to achieve consensus.

### Statistical analysis

The current meta-analysis was performed by using STATA v16.0 (StataCorp, US). I^2^ statistic and Q test were conducted to assess between-study heterogeneity. *I*^2^ < 50% and Q test *P* > 0.10 indicated low level of heterogeneity. Yet, we still used a random-effect model for all analyses regardless of between-study heterogeneity to gain a more conservative pooled estimates than using a fixed-effect model. The relative risk (RR) and corresponding 95% confidence interval (CI) were calculated to estimate the risk of categorical outcomes comparing MLP group to LP group. Meanwhile, the mean difference (MD) with 95%CI was estimated for continuous variables. Subgroup analysis regarding fracture type (Gartland type III only, various types) and use of a mini-open technique were performed in meta-analysis including 10 or more eligible studies. For loss of reduction, additional subgroup analysis that allowing a 3^rd^ pin if required was performed. Moreover, sensitivity analysis was conducted to judge whether a single study significantly influences the overall pooled estimates by applying the Leave-One-Out method. Publication bias of meta-analysis with 10 or more eligible studies was assessed by viewing the symmetry of funnel plot and Egger’ test. The funnel plot, if asymmetrical, was trim-and-filled by imputing hypothetical negative unpublished studies to see whether publication bias significantly influenced the effect estimates. *P* value < 0.05 was considered statistically significant.

The certainty of evidence of pooled effect estimate of main outcomes was assessed by using the Grading of Recommendations Assessment, Development and Evaluation (GRADE) approach [21]. An overall certainty was graded according to assessment of domains of risk of bias, inconsistency, indirectness, imprecision, and publication bias. Two authors performed the assessment.

## Results

### Characteristics of included studies

**Fig. 1:**
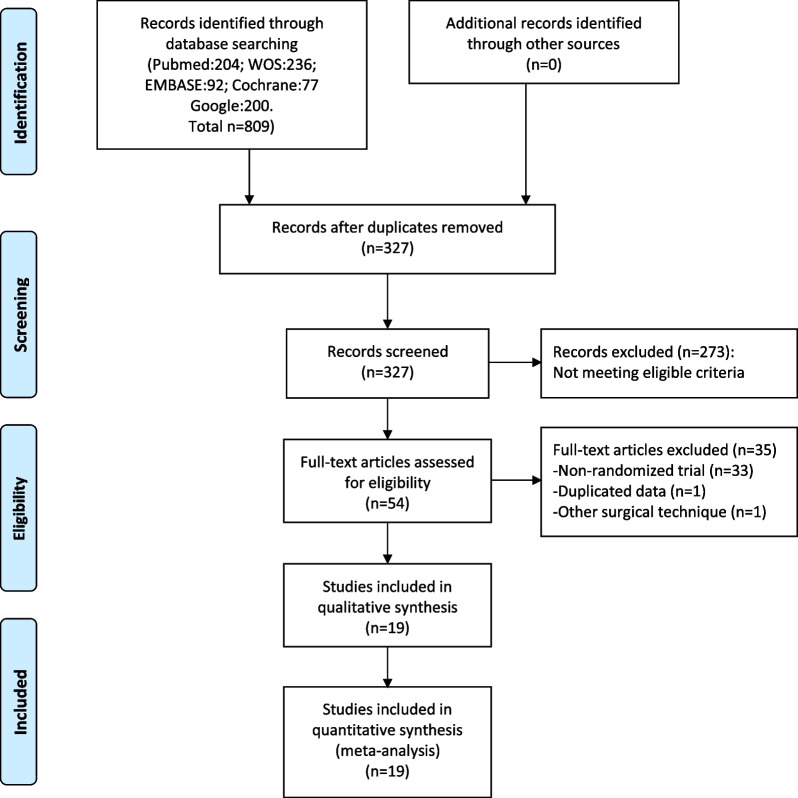
Flowchart of literature search and selection

The literature search identified a total of 327 unique articles after removing duplicates. Finally, 19 RCTs comprising 1297 children with SCHF were included in current meta-analysis [22–40] (Fig. 1). There were 648 patients assigned to MLP group and 649 assigned to LP group. Ten studies recruited only Gartland type III fractures, eight enrolled both Gartland type II and III fractures, and one included Gartland type II, III and IV fractures [23]. Six RCTs allowed the use of a third pin if required [23, 25, 28, 29, 34, 36], while seven trials used a medial mini-open technique [22, 24, 25, 27, 30, 37, 38]. The follow-up widely ranged from 1.8 to 11.43 months. The baseline characteristics of included trials are summarized in Table 1.

**Table 1:** Baseline characteristics of studies included in meta-analysis

References	Sample size		Mean age, years		Gender (male/female)		Gartland type	Follow-up, months	
	MLP	LP	MLP	LP	MLP	LP		MLP	LP
Foead et al. [31]	34	32	NA	NA	NA	NA	II/III	NA	NA
Kocher et al. [30]	24	28	5.7	6.1	13/11	10/18	III	3	3
Tripuraneni et al. [29]	20	20	5.5	4.3	NA	NA	II/III	1.8	2.2
Gaston et al. [28]	57	47	6.2	5.7	31/26	22/25	III	NA	NA
Anwar et al. [32]	25	25	NA	NA	NA	NA	II/III	6	6
Maity et al. [27]	80	80	6.24	6.12	48/32	51/29	II/III	3	3
Shah and Arif [33]	100	100	6.51	5.83	80/20	78/22	II/III	3	3
Abdel Karim et al. [26]	30	30	NA	NA	NA	NA	II/III	3	3
Prashant et al. [25]	31	31	8.55	8.25	22/9	23/8	III	8.4	8.8
Naik et al. [24]	29	28	6.28	7.2	20/9	16/12	III	5.7	5.9
Naveen and Chaitanya [34]	20	20	7.37	7.62	12/8	13/7	II/III	6	6
Patil et al. [35]	15	15	NA	NA	10/5	9/6	III	6	6
Arun et al. [36]	30	38	NA	NA	NA	NA	II/III	6	6
Afaque et al. [23]	40	37	7.2	6.8	27/13	21/16	II/III/IV	3	3
Abubeih et al. [37]	33	34	5.4	4.9	24/9	21/13	III	3	3
Palange et al. [38]	15	15	6.52	7.41	NA	NA	III	11.43	10.36
Othman and Hamawand [39]	15	15	7.02	6.44	11/4	9/6	III	2.4	3.1
Natalin et al. [22]	19	24	NA	NA	NA	NA	III	6	6
Singh et al. [40]	31	30	8.54	8.26	23/8	22/8	III	8.4	8.8

*MLP* medial–lateral entry pinning, *LP* lateral entry pinning, *NA* not available

### Risk of bias assessment

Four trials had high risk of bias of random sequence generation, as two assigned patients according to which surgeon was on call [28, 29] and the other two according to odd and even inpatient numbers [24, 39]. Five RCTs without reporting the randomization procedure were considered to have unclear risk, and the other ten trials had low risk of such bias. Five studies reported proper allocation concealment [26, 27, 30, 37, 38]. Patients in three trials [26, 27, 30] and researchers assessing outcomes in six trials [22, 26, 27, 30, 34, 40] were not blinded to interventions. Thus, they had a high risk of performance or detection bias. Over a half of included trials reported a certain proportion of loss of follow-ups without detailly describing the reasons, and the other nine studies had no loss of follow-ups. All RCTs, except Naveen et al.’s [34], had unclear risk of reporting bias. The other bias of all studies was of unclear risk. Overall, there was obvious risk of bias in most of included studies (Additional file 2: Figs. S1 and S2).

### Radiographical outcomes

**Fig. 2:**
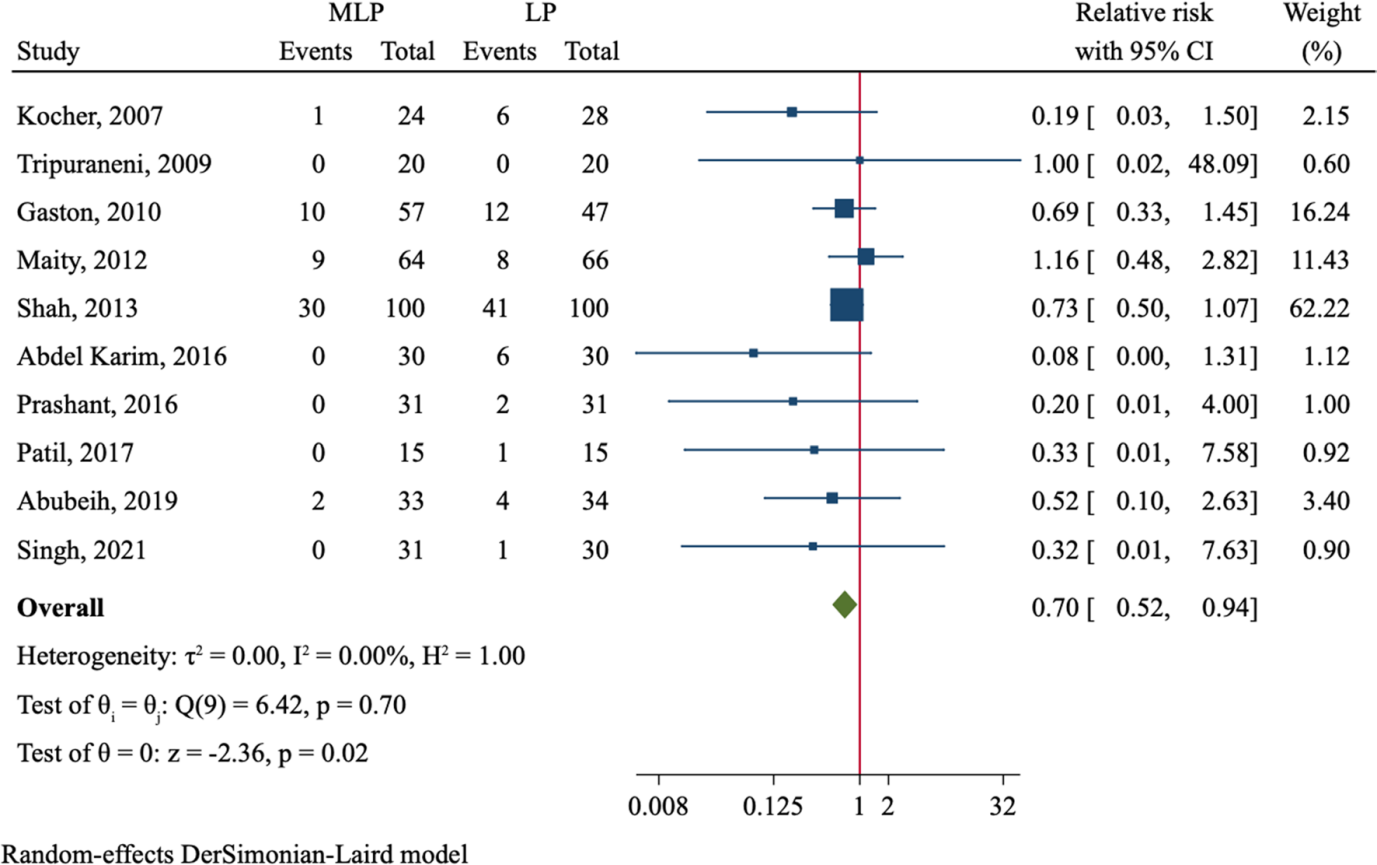
Comparison of loss of reduction between medial–lateral entry group and lateral entry group

Loss of reduction was reported in ten studies comprising 806 patients (Table 2). Loss of reduction occurred in 52 (12.8%) of 405 fractures using crossed pin fixation and 81 (20.2%) of 401 fractures using lateral pin fixation. The incidence of loss of reduction in MLP group was lower than that of LP group with significant difference (RR = 0.70, 95%CI 0.52–0.94, *P* = 0.018, Fig. 2). We further divided the analysis into several subgroups (Table 2). In patients with Gartland type III fractures, MLP reduced the risk of loss of reduction (RR = 0.53, 95%CI 0.29–0.98, *P* = 0.041). In addition, patients receiving MLP without using a mini-open technique had a lower risk of loss of reduction (RR = 0.70, 95%CI 0.50–0.98, *P* = 0.035). We did not find significant association of pin configuration with risk of loss of reduction in subgroups allowing or not allowing a 3rd pin.

**Table 2:** Results of subgroup analyses

Variable	No. of studies	No. of patients (MLP/LP)	Effect estimates	95%CI	*P*	Heterogeneity	
						*I*^2^ (%)	*P*
Loss of Baumann angle	10	348/340	MD = 0.08	− 0.15, 0.30	0.507	0	0.688
Gartland type III only	6	191/185	MD = 0.02	− 0.36, 0.40	0.905	13.3	0.330
Various Gartland types	4	157/155	MD = 0.09	− 0.21, 0.39	0.563	0	0.866
With mini-open technique	3	121/128	MD = -0.41	− 0.99, 0.17	0.168	0	0.583
Without mini-open technique	7	227/212	MD = 0.16	− 0.08, 0.40	0.197	0	0.889
Loss of reduction	10	405/401	RR = 0.70	0.52–0.94	0.018	0	0.697
Gartland type III only	6	191/185	RR = 0.53	0.29–0.98	0.041	0	0.853
Various Gartland types	4	214/216	RR = 0.77	0.48–1.25	0.228	12.8	0.329
With mini-open technique	4	151/158	RR = 0.48	0.16–1.44	0.191	42.5	0.157
Without mini-open technique	6	254/243	RR = 0.70	0.50–0.98	0.035	0	0.944
3rd pin allowed	4	123/113	RR = 0.63	0.31–1.26	0.189	0	0.842
2 pins only	6	282/288	RR = 0.69	0.45–1.07	0.095	13.4	0.329
Flynn criteria, excellent	15	491/506	RR = 1.06	0.99–1.14	0.102	0	0.995
Gartland type III only	9	212/220	RR = 1.06	0.95–1.18	0.274	0	0.988
Various Gartland types	6	279/286	RR = 1.07	0.96–1.18	0.223	0	0.810
With mini-open technique	6	184/195	RR = 1.06	0.95–1.19	0.323	0	0.947
Without mini-open technique	9	307/311	RR = 1.07	0.97–1.18	0.191	0	0.945
Iatrogenic ulnar nerve injury	18	542/544	RR = 2.21	1.11–4.41	0.024	0	0.999
Gartland type III only	10	269/267	RR = 2.36	0.89–6.29	0.085	0	0.983
Various Gartland types	8	273/277	RR = 2.07	0.70–5.48	0.141	0	0.963
With mini-open technique	7	230/239	RR = 1.73	0.47–6.31	0.407	0	0.856
Without mini-open technique	11	312/305	RR = 2.44	1.08–5.51	0.032	0	0.999
Pin tract infection	12	362/363	RR = 0.92	0.50–1.70	0.798	0	0.988
Gartland type III only	6	144/149	RR = 0.95	0.36–2.48	0.914	0	0.862
Various Gartland types	6	218/214	RR = 0.91	0.41–2.00	0.809	0	0.940
With mini-open technique	4	162/168	RR = 1.03	0.37–2.87	0.950	0	0.946
Without mini-open technique	8	200/195	RR = 0.87	0.40–1.86	0.715	0	0.910

*MLP* medial–lateral entry pinning, *LP* lateral entry pinning, *MD angle* metaphysio-diaphyseal angle, *HC angle* humerocapitellar angle, *MD* mean difference, *RR* relative risk, *CI* confidence interval

The loss of carrying angle was compared between MLP and LP groups in seven RCTs, and the loss of Baumann angle was compared in 10 trials (Table 2). There was no statistically significant difference between both percutaneous pin fixation methods in terms of loss of carrying angle (MD = − 0.12, 95%CI − 0.39 to 0.16, *P* = 0.415, Additional file 2: Fig. S3) and loss of Baumann angle (MD = 0.08, 95%CI − 0.15 to 0.30, *P* = 0.507, Additional file 2: Fig. S4). Similarly, we observed no difference of the other radiographic outcomes including carrying angle, Baumann angle, loss of HC angle, and loss of MD angle (Additional file 2: Fig. S5).

### Functional outcomes

**Fig. 3:**
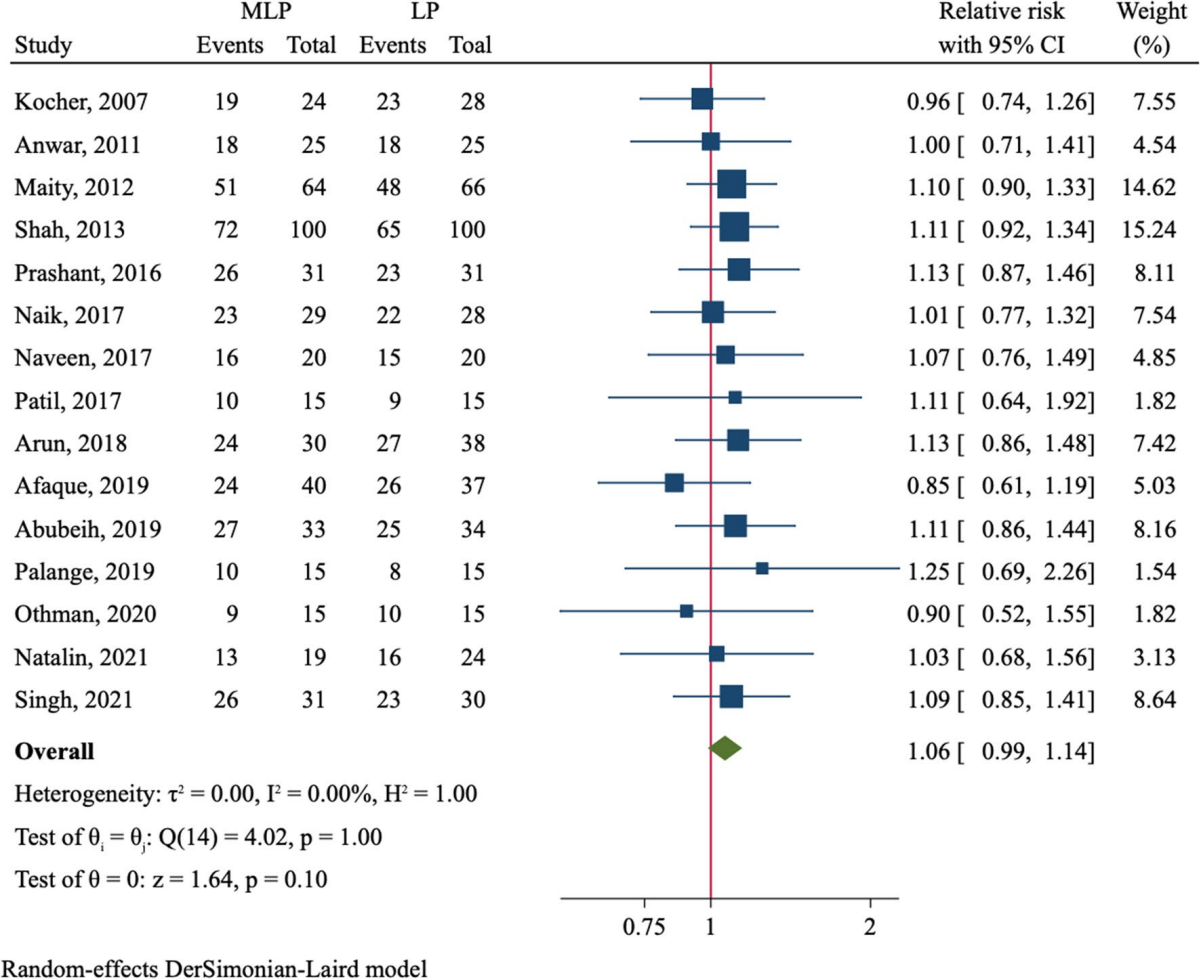
Comparison of excellent grading of Flynn criteria between medial–lateral entry group and lateral entry group

The functional outcome according to Flynn criteria was assessed in 15 trials (Table 2). In MLP group, 74.9% (368/491) of patients reported an excellent outcome, which was similar to 70.8% (358/506) in LP group (RR = 1.06, 95%CI 0.99–1.14, *P* = 0.102, Fig. 3). No significant difference was observed among all subgroups in terms of fracture type and use of mini-open technique (Table 2). The other outcomes, including loss of elbow extension, loss of elbow flexion and loss of range of motion, did not differ between both groups (Additional file 2: Fig. S5).

### Complications

**Fig. 4:**
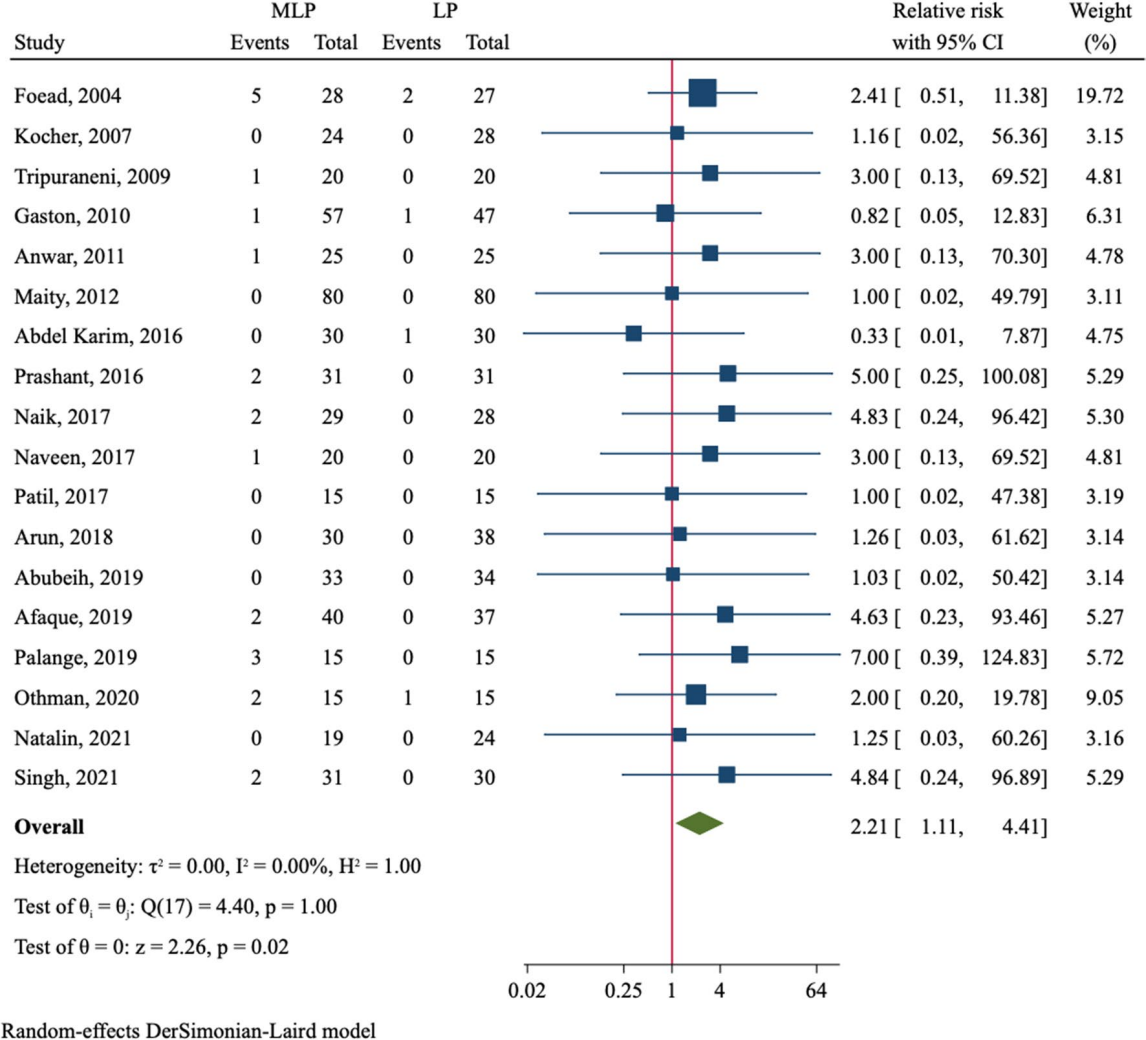
Comparison of iatrogenic ulnar nerve injury between medial–lateral entry group and lateral entry group

**Fig. 5:**
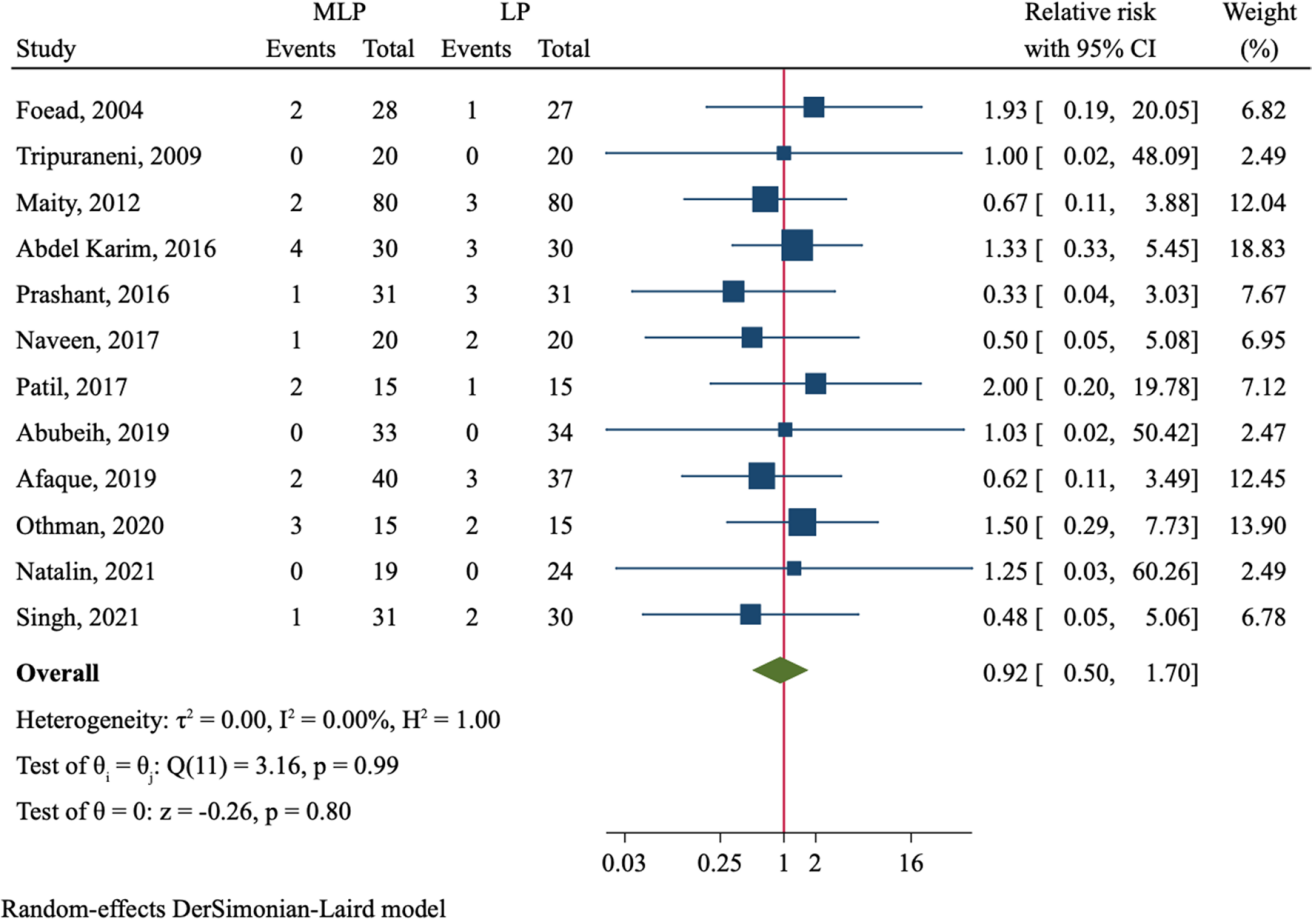
Comparison of pin tract infection between medial–lateral entry group and lateral entry group

The incidence of iatrogenic ulnar nerve injury was compared in 18 RCTs (Table 2). Six studies observed no injury in all patients [22, 27, 30, 35–37]. Among 542 fractures with crossed medial–lateral pinning, there were 22 cases suffering injury of iatrogenic ulnar nerve. Of 544 patients treated with lateral pinning, only five had ulnar nerve injury. The incidence of iatrogenic ulnar nerve injury was 4.1% in MLP group as compared to 0.9% in LP group. Therefore, crossed entry pin fixation conferred a significant higher risk of iatrogenic ulnar nerve injury than lateral entry pin configuration (RR = 2.21, 95%CI 1.11–4.41, *P* = 0.024, Fig. 4). We furtherly divided the analysis by the use of a mini-open technique which could help to avoid injury of ulnar nerve in crossed entry pinning (Additional file 2: Fig. S6). Among seven studies using the mini-open technique [22, 24, 26, 27, 30, 37, 38], only 2.2% (5/230) of patients in MLP group suffered ulnar nerve injury, which did not differ from that in LP group (RR = 1.73, 95%CI 0.47–6.31, *P* = 0.407). However, in patients treated with crossed pin fixation but without using the mini-open technique, the incidence of ulnar nerve injury was 5.4% (17/312), which was significantly higher than 1.3% (4/305) in LP group (RR = 2.44, 95%CI 1.08–5.51, *P* = 0.032). As to pin tract infection, the incidence was 5.0% (18/362) and 5.5% (20/363) in MLP group and LP group, respectively, which were not significantly different (RR = 0.92, 95%CI 0.50–1.70, *P* = 0.798, Fig. 5).

### Sensitivity analysis and publication bias

Sensitivity analysis showed that Shah et al.’s study [33] significantly influenced the pooled OR estimate of loss of reduction. This study had an exceptionally high rate of loss reduction [33]. After exclusion, MLP was no longer significantly associated with risk of loss of reduction (RR = 0.64, 95%CI 0.39–1.05, *P* = 0.076). For the other analyses, the pooled results were not significantly changed regardless of which study was omitted.

We assessed the publication bias of the meta-analyses of loss of Baumann angle, loss of reduction, excellent outcome of Flynn criteria, iatrogenic ulnar nerve injury and pin tract infection, each of which included 10 or more studies. An asymmetric funnel plot was found in analysis of loss of reduction, and Egger’s test indicated potential bias (*P* = 0.117). After trim-and-fill analysis by imputing four studies, the association was not statistically significant any more (RR = 0.75, 95%CI 0.56–1.01, Additional file 2: Fig. S7). For the other meta-analysis, the funnel plots were symmetric (Additional file 2: Fig. S7) and Egger’s tests indicated no obvious evidence of publication bias (*P* > 0.05).

### Certainty of evidence

According to GRADE approach (Table 3), the certainty of evidence of pooled effect estimate is graded as very low in analyses of loss of reduction, iatrogenic ulnar nerve injury and pin tract infection. The certainty of evidence is considered as low in analyses of excellent Flynn criteria, loss of Baumann angle and loss of carrying angle.

**Table 3:** Summary of the certainty of evidence using GRADE approach

No. of studies (participants)	Risk of bias	Inconsistency	Indirectness	Imprecision	Publication bias	Certainty of evidence
Loss of reduction, 10 RCTs (806)	Serious^a^	Not serious	Serious^b^	Serious^c,d^	Suspected^e^	⊕◯◯◯ Very low
Excellent Flynn criteria, 15 RCTs (997)	Serious	Not serious	Serious^b^	Not serious	None	⊕⊕◯◯ Low
Iatrogenic ulnar nerve injury, 18 RCTs (1086)	Serious	Not serious	Serious^b^	Serious^c,d^	None	⊕◯◯◯ Very low
Pin tract infection, 12 RCTs (725)	Serious	Not serious	Serious^b^	Serious^c,d^	None	⊕◯◯◯ Very low
Loss of Baumann angle, 10 RCTs (688)	Serious	Not serious	Serious^b^	Not serious	None	⊕⊕◯◯ Low
Loss of carrying angle, 7 RCTs (465)	Serious	Not serious	Serious^b^	Not serious	None	⊕⊕◯◯ Low

^a^The majority of included studies had a high risk of bias

^b^Studies using mini-open technique or allowing a 3rd pin were included

^c^The 95% confidence interval is wide

^d^The number of events is small

^e^Potential small-study effect

## Discussion

There have been agreements on the comparable functional and radiographic outcomes between media-lateral pinning and lateral-only pinning for displaced supracondylar fractures in children, but the optimal choice of pin configuration regarding two major complications, i.e., iatrogenic ulnar nerve injury and loss of fixation, is still in controversy. The present meta-analysis, incorporating evidence from 19 available RCTs, demonstrates that medial–lateral pinning fixation has a higher risk of iatrogenic ulnar nerve injury but a lower incidence of loss of reduction than lateral-only pinning fixation. The radiographical outcomes with respect to carrying angle, loss of carrying angle, Baumann angle, loss of Baumann angle, loss of HC angle, loss of MD angle, and functional outcomes including Flynn criteria scores, loss of elbow extension, loss of elbow flexion, loss of range of motion, and the complication of pin tract infection are all similar for both pinning fixations. Moreover, the mini-open technique reduces the risk of ulnar nerve injury of medial–lateral pin configuration, which is shown to be as safe as the lateral-only pinning.

Despite an increased risk of iatrogenic ulnar nerve injury in crossed pinning fixation, the overall incidence of this complication is still very low. In our study, the injury occurs in 4.1% of patients in MLP group and 0.9% in LP group, which is similar to previous studies [9, 10]. The incidence of ulnar nerve injury in MLP group can be greatly reduced by using a mini-open technique [17, 41]. The mini-open technique is to make a small incision at the medial epicondyle and explore the ulnar nerve prior to medial pin placement to avoid nerve injury. Subgroup analysis shows the overall incidence of injury reduces to 2.2% (5/230) in trials using the mini-open technique, which is not different from that of LP group. Conversely, the injury risk of MLP group in trials without using the mini-open technique is still significantly higher than that of LP group. The major drawback of mini-open technique is that it may take a longer duration for surgery and leave a surgical scar [24]. These results suggest that crossed pinning fixation with a mini-open technique is an effective and safe treatment strategy for SCHFs. However, we noticed there were still some cases of ulnar nerve injury in the LP group [26, 28, 31, 39], in which the lateral pin is unlikely to be the cause as the pin is not inserted anywhere near the ulnar nerve. Although the cause is still unclear, either over drilling or over traction during manipulation may attribute to the ulnar nerve injury in LP cases [26].

The disadvantage of lateral entry pinning is the increased risk of loss of reduction, which may result in cubitus varus and additional surgery, due to less biomechanical stability than medial–lateral entry pinning. Several meta-analyses incorporating only high level-of-evidence RCTs, as well as the present one, have found higher chances of loss of reduction in lateral entry pinning group [7, 12, 13]. The lateral pins are usually inserted in a divergent or parallel manner. Biomechanical analyses show that divergent pins have a stronger stability than parallel pins and a comparable stability to medial–lateral crossed pins [8, 42]. Besides, the placement of a 3rd lateral pin may also provide a satisfactory stability if necessary [43, 44]. In the setting of three lateral pins, both of the divergent and parallel configurations can achieve adequate stability without significant difference [45]. In present study, subgroup analysis regarding divergent or parallel configuration of lateral pins is not conducted due to insufficient data. However, we did not find significant association for risk of loss reduction when allowing a 3rd pin or not, which may be due to a relatively small sample size.

Our analysis has some limitations. The first one is the poor methodological quality. The majority of included RCTs have unclear or high risk of selection, performance, detection, attrition and reporting bias. Secondly, each of the included trials has a relatively small number of participants and the overall sample size of our meta-analysis is limited. Thus, the statistical power may not be adequate, and the results may be not robust enough. Thirdly, there is high clinical heterogeneity in terms of duration of follow-ups, fracture type, number of pins, clinical experience level of surgeons and delay of surgery, which may have impact on the clinical outcomes. Fourthly, Gartland II fractures can be divided into IIA and IIB according to the modified Gartland classification [46], and type IIA fractures are rotationally stable than type IIB fractures, which may introduce some bias to the analysis of loss of reduction. Yet, we could not perform such a subgroup analysis as the data were not available. More registered, well-designed, large-scaled RCTs are needed in the future.

## Conclusions

Taken together, our meta-analysis suggests that medial–lateral crossed pinning fixation with a mini-open technique is an effective and safe strategy for the management of children with displaced supracondylar humeral fractures.

## Supplementary Information

**Additional file 1.** PRISMA Checklist.

**Additional file 2: Fig. S1.** Risk of bias summary. **Fig. S2.** Risk of bias graph. **Fig. S3.** Comparison of loss of carrying angle between medial–lateral entry group and lateral entry group. **Fig. S4.** Comparison of loss of Baumann angle between medial–lateral entry group and lateral entry group. **Fig. S5.** Comparison of carrying angle, Baumann angle, loss of elbow extension loss, loss of elbow flexion, loss of humerocapitellar angle, loss of metaphysio-diaphyseal (MD) angle, and loss of range of motion between medial–lateral entry group and lateral entry group. **Fig. S6.** Subgroup analysis of iatrogenic ulnar nerve injury divided by the use of mini-open technique in crossed entry group. **Fig. S7.** Funnel plots for meta-analyses including 10 or more studies. **A** Trim-and-filled plot for loss of reduction. **B** Loss of Baumann angle. **C** Excellent grading of Flynn criteria. **D** Iatrogenic ulnar nerve injury. **E** Pin tract infection.

## Data Availability

All data generated or analyzed during this study are included in this published article and its supplementary information files.

## Abbreviations

SCHFs: Supracondylar humeral fractures
MLP: Medial–lateral crossed entry pinning
LP: Lateral-only entry pinning
RCT: Randomized controlled trial
RR: Risk ratio
