# Supplementary material for: Medial–lateral versus lateral-only pinning fixation in children with displaced supracondylar humeral fractures: a meta-analysis of randomized controlled trials
Source: J Orthop Surg Res. 2023 Jan 16;18:43. doi: 10.1186/s13018-023-03528-8 (PMC9841617; doi:10.1186/s13018-023-03528-8)
Supplement: Supplementary file 2 — Additional file 2: Fig. S1. Risk of bias summary. Fig. S2. Risk of bias graph. Fig. S3. Comparison of loss of carrying angle between medial–lateral entry group and lateral entry group. Fig. S4. Comparison of loss of Baumann angle between medial–lateral entry group and lateral entry group. Fig. S5. Comparison of carrying angle, Baumann angle, loss of elbow extension loss, loss of elbow flexion, loss of humerocapitellar angle, loss of metaphysio-diaphyseal (MD) angle, and loss of range of motion between medial–lateral entry group and lateral entry group. Fig. S6. Subgroup analysis of iatrogenic ulnar nerve injury divided by the use of mini-open technique in crossed entry group. Fig. S7. Funnel plots for meta-analyses including 10 or more studies. A Trim-and-filled plot for loss of reduction. B Loss of Baumann angle. C Excellent grading of Flynn criteria. D Iatrogenic ulnar nerve injury. E Pin tract infection. [file 13018_2023_3528_MOESM2_ESM.docx]

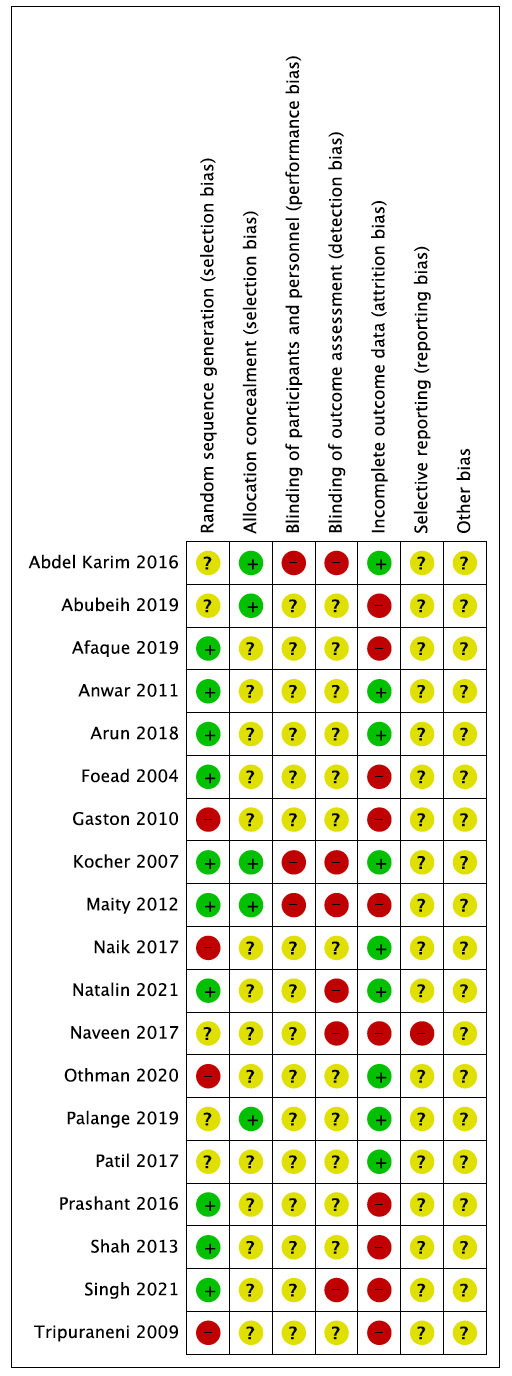


Figure S1 Risk of bias summary.


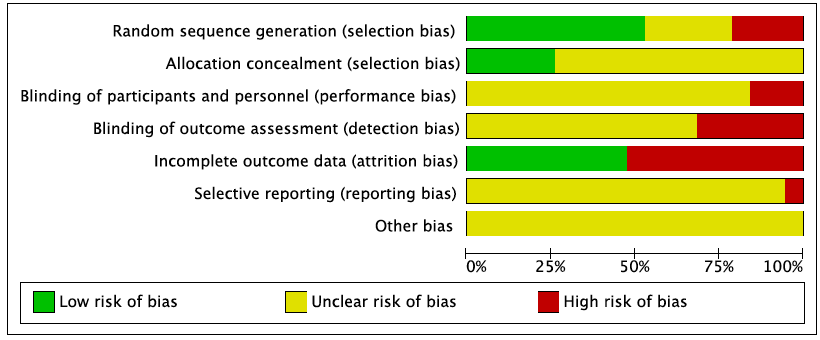


Figure S2 Risk of bias graph.


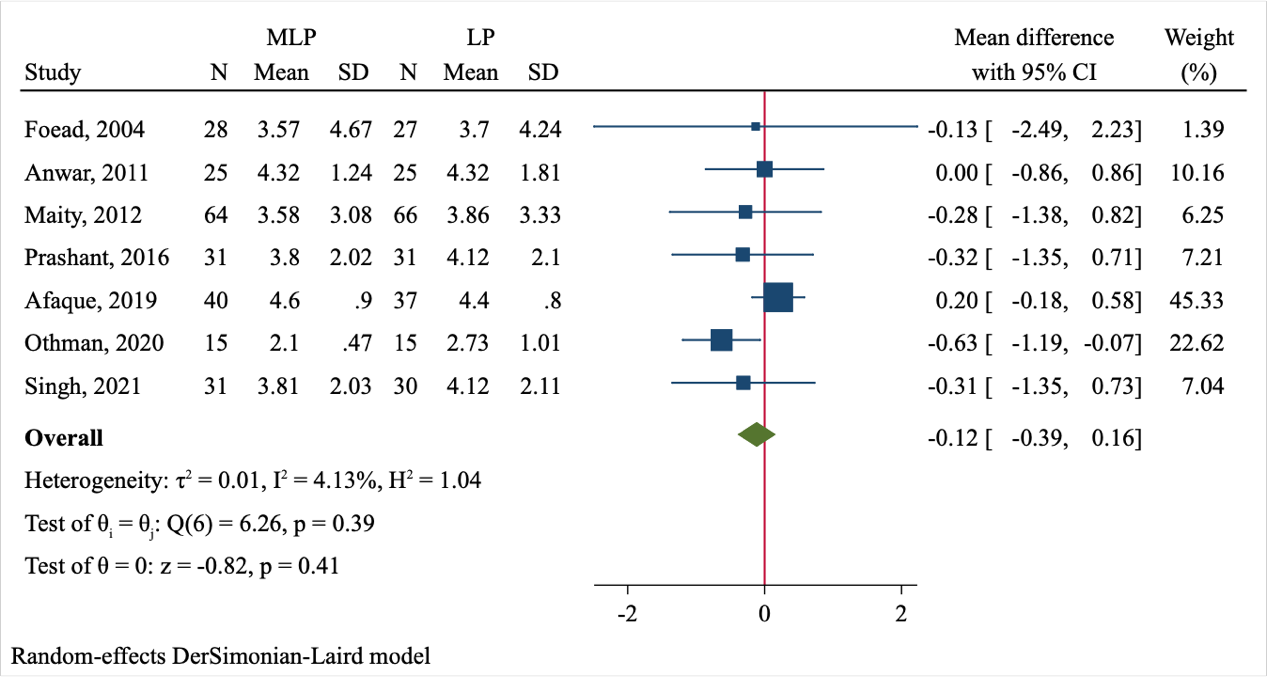


Figure S3 Comparison of loss of carrying angle between medial-lateral entry group and lateral entry group.


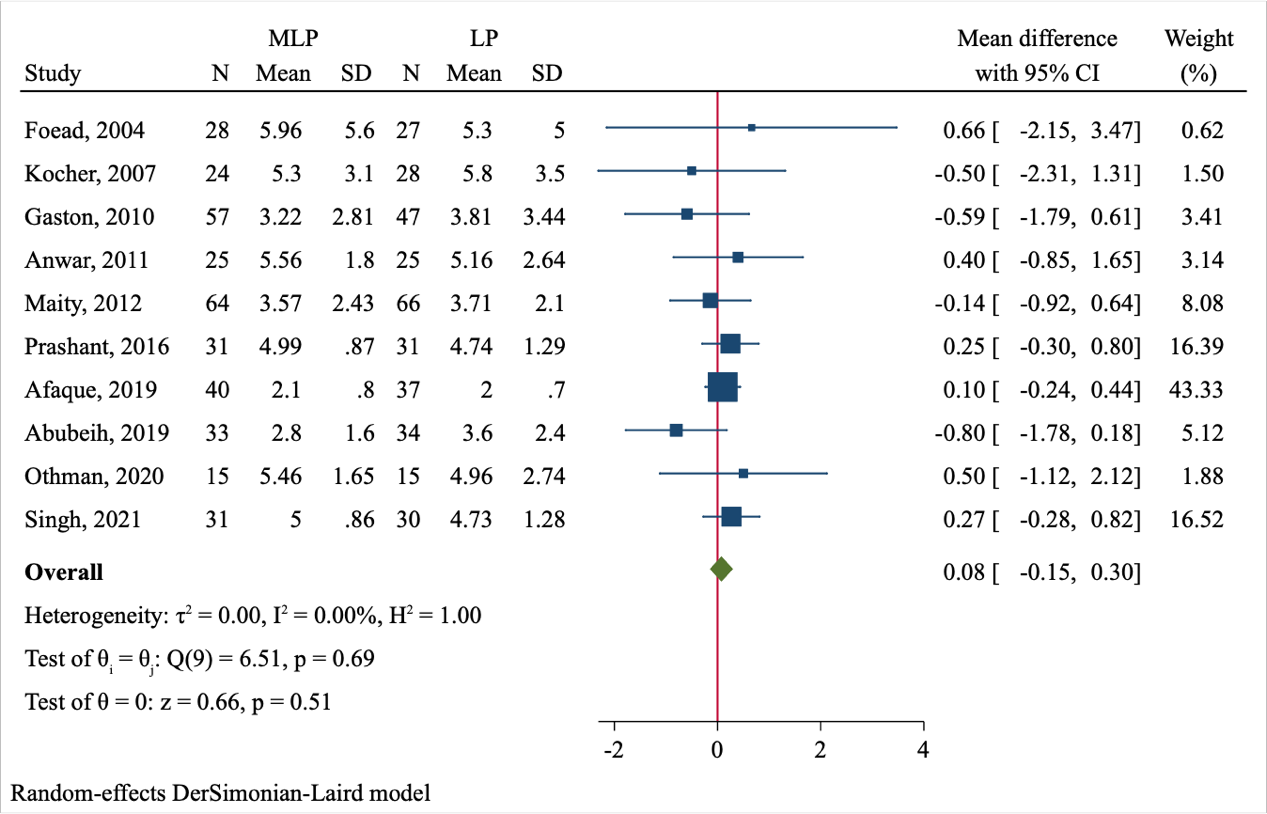


Figure S4 Comparison of loss of Baumann angle between medial-lateral entry group and lateral entry group.


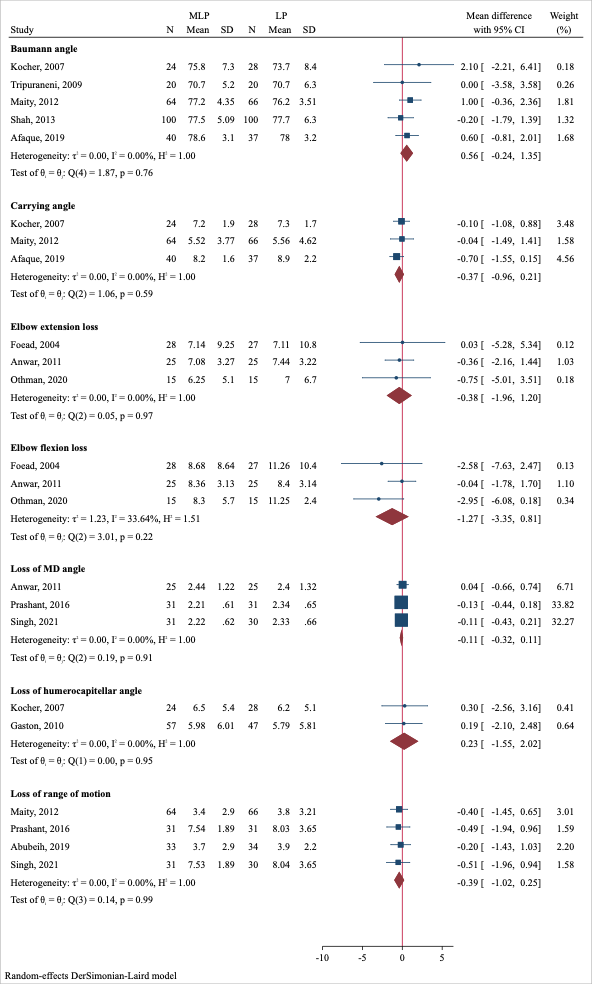


Figure S5 Comparison of carrying angle, Baumann angle, loss of elbow extension loss, loss of elbow flexion, loss of humerocapitellar angle, loss of metaphysio-diaphyseal (MD) angle, and loss of range of motion between medial-lateral entry group and lateral entry group.


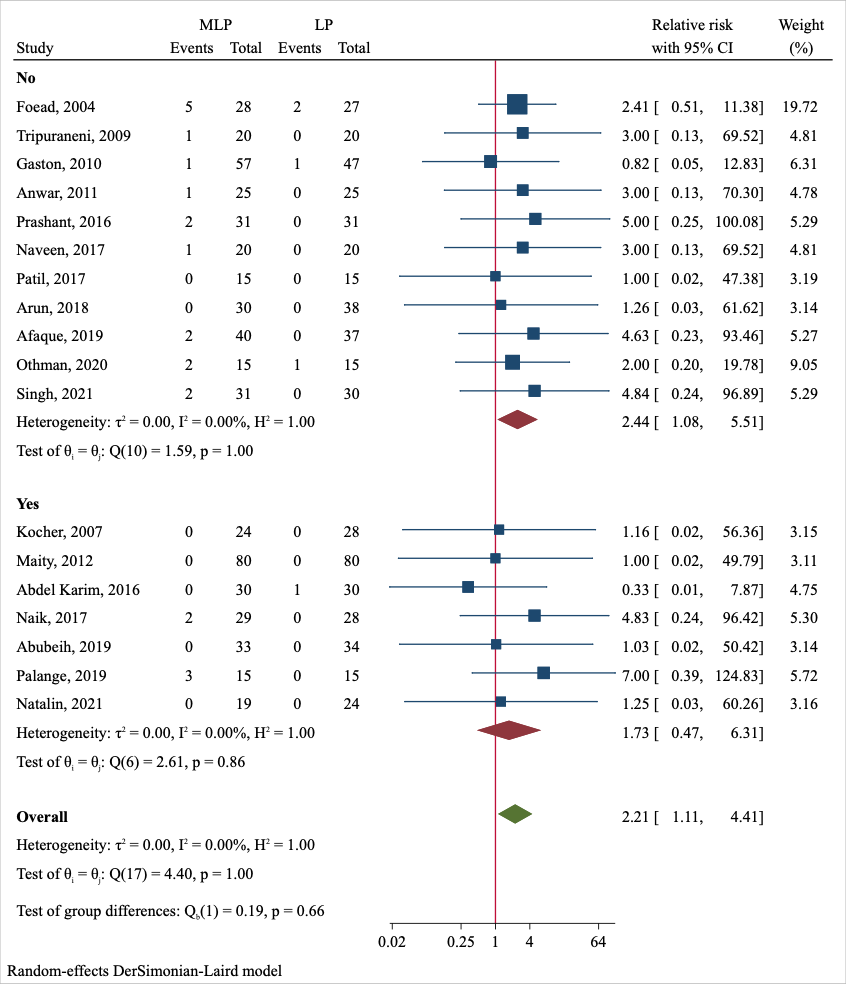


Figure S6 Subgroup analysis of iatrogenic ulnar nerve injury divided by the use of mini-open technique in crossed entry group.


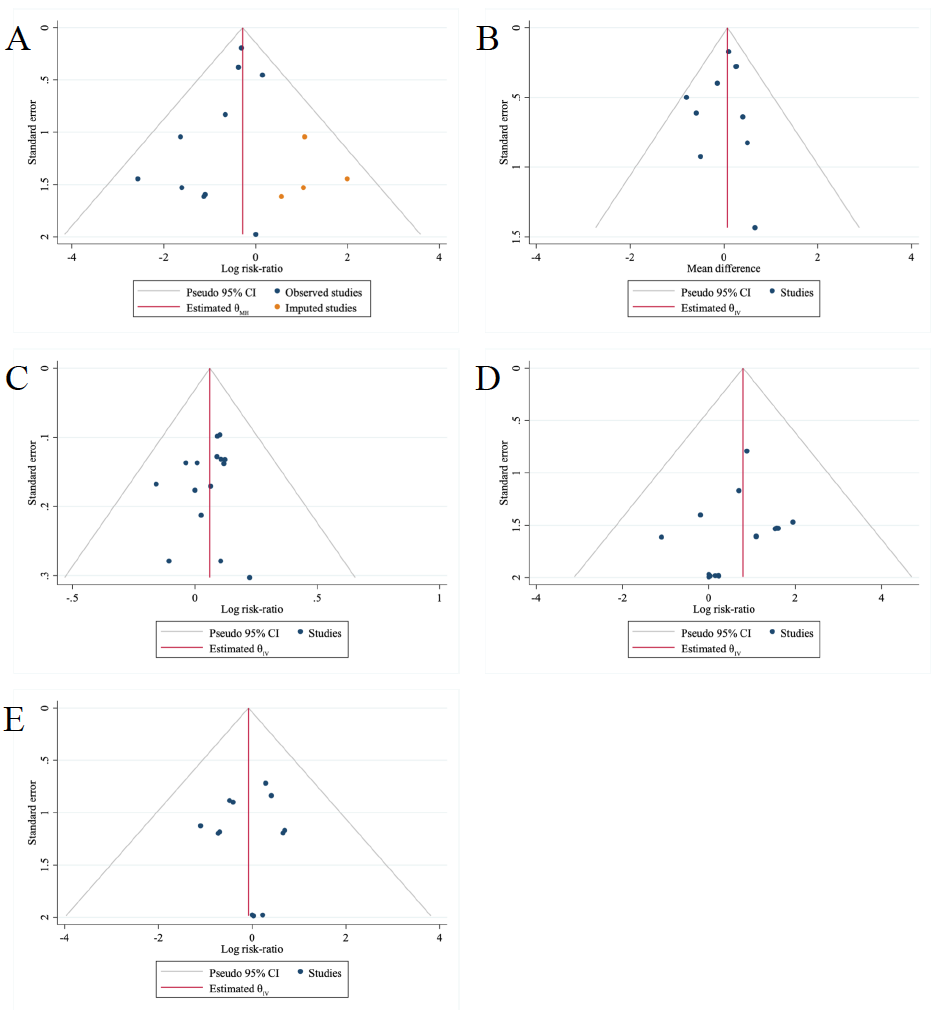


Figure S7 Funnel plots for meta-analyses including 10 or more studies. (A) Trim-and-filled plot for loss of reduction. (B) Loss of Baumann angle. (C) Excellent grading of Flynn criteria. (D) Iatrogenic ulnar nerve injury. (E) Pin tract infection.
